# Anti-malarial prescriptions in three health care facilities after the emergence of chloroquine resistance in Niakhar, Senegal (1992–2004)

**DOI:** 10.1186/1475-2875-8-83

**Published:** 2009-04-27

**Authors:** Aline Munier, Aldiouma Diallo, Michel Cot, Ousmane Ndiaye, Pascal Arduin, Jean-Philippe Chippaux

**Affiliations:** 1 Unité de recherche Santé de la mère et de l'enfant en milieu tropical, IRD UR010, Université Paris Descartes, 4 av de l'Observatoire, 75270 Paris cedex 06, France; 2 Unité de recherche Suivi démographique, épidémiologique et environnemental, IRD US009, Dakar, BP 1386, Sénégal

## Abstract

**Background:**

In the rural zone of Niakhar in Senegal, the first therapeutic failures for chloroquine (CQ) were observed in 1992. In 2003, the national policy regarding first-line treatment of uncomplicated malaria was modified, replacing CQ by a transitory bi-therapy amodiaquine/sulphadoxine-pyrimethamine (AQ/SP), before the implementation of artemisinin-based combination therapy (ACT) in 2006.

The aims of the study were to assess the evolution of anti-malarial prescriptions in three health care facilities between 1992 and 2004, in parallel with increasing CQ resistance in the region.

**Methods:**

The study was conducted in the area of Niakhar, a demographic surveillance site located in a sahelo-sudanese region of Senegal, with mesoendemic and seasonal malaria transmission. Health records of two public health centres and a private catholic dispensary were collected retrospectively to cover the period 1992–2004.

**Results:**

Records included 110,093 consultations and 292,965 prescribed treatments. Twenty-five percent of treatments were anti-malarials, prescribed to 49% of patients. They were delivered all year long, but especially during the rainy season, and 20% of patients with no clinical malaria diagnosis received anti-malarials. Chloroquine and quinine represented respectively 55.7% and 34.6% of prescribed anti-malarials. Overall, chloroquine prescriptions rose from 1992 to 2000, in parallel with clinical malaria; then the CQ prescription rate decreased from 2000 and was concomitant with the rise of SP and the persistence of quinine use. AQ and SP were mainly used as bi-therapy after 2003, at the time of national treatment policy change.

**Conclusion:**

The results show the overall level of anti-malarial prescription in the study area for a considerable number of patients over a large period of time. Even though resistance to CQ rapidly increased from 1992 to 2001, no change in CQ prescription was observed until the early 2000s, possibly due to the absence of an obvious decrease in CQ effectiveness, a lack of therapeutic options or a blind follow-up of national guidelines.

## Background

One of the main issues in worldwide management of malaria is the occurrence of chemoresistance and its rapid spread. Efforts to control and treat malaria have already been seriously compromised, particularly by the resistance of *Plasmodium falciparum *to chloroquine (CQ), then to other drugs, such as sulphadoxine-pyrimethamine (SP), mefloquine, and amodiaquine (AQ). Resistance to chloroquine first appeared in South-East Asia (Thai-Cambodian border) and in South America (Colombia and Venezuela) in the late 1950s [1,2]. In Africa, it was first reported in Kenya and Tanzania in 1978 [3-6]. It then spread to other countries and to Centre and South Africa [7-12], before reaching West Africa in 1983 [13-15]. By 1989, resistance was widespread in sub-Saharan Africa.

In the area of Niakhar in Senegal, first therapeutic failures to CQ were observed in 1992 and chemoresistance then progressively increased [16,17]. The public health impact of chloroquine resistance was shown in many parts of Africa [18] and a rise in malaria specific mortality, measured by verbal autopsy, was demonstrated in three rural regions of Senegal (Mlomp, Bandafassi and Niakhar)[19], where demographic and epidemiological surveillance was particularly well developed. In Niakhar, a two-fold increase in malaria mortality among children 0–9 years was shown, from 4.0 to 8.2 per 1,000 children (0–9) per year between the periods (1984–1991) and (1992–1995).

However, national policy concerning malaria treatment did not change until 2003. A consensus conference held by the Ministry of Health in June 2003 led to the replacement of CQ by the transitory bi-therapy AQ/SP[20], before the implementation of artemisinin-based combination therapy (ACT) using artesunate/amodiaquine (AS/AQ) in 2006.

Data discussed in the present article concern a considerable number of patients and prescriptions in three rural health care facilities over a long period, from the emergence of CQ resistance in 1992, until the treatment policy change in 2003 and its application in health centres. An attempt was made to assess the evolution of therapeutic practices in peripheral health care facilities according to the period of study and the dispensary.

The aim of the research was to study the evolution of anti-malarial prescriptions in Niakhar area in the context of the increase in CQ resistance estimated by surveys realized in the region during the same period.

## Methods

### Study area and population

#### Niakhar study area

The study was conducted in the area of Niakhar, a sahelo-sudanese zone located in the region and department of Fatick, 130 kilometres south-east of Dakar, Senegal (N 14°30, W 16°30). A demographic surveillance site has been implemented in this area in 1963 by the Institut de Recherche pour le Développement (IRD, formerly ORSTOM) and is currently composed of 30 villages [21]. The zone covers 230 km^2^. It had 26,356 inhabitants on the 1^st ^January 1992 and 33,890 on the 1^st ^January 2004, mostly from the Sereer ethnic group and Muslim religion. Malaria transmission in the area is endemic and occurs principally from August to November, following the rainy season from July to October.

#### Health care facilities

Three health care facilities are located in the villages of Diohine, Toucar and Ngayokhem, which are among the most populated villages of the area (3,311, 3,686 and 2,332 inhabitants in 2004 respectively). Patient management is carried out in Diohine by a private catholic dispensary, in Toucar and Ngayokhem by public health centres. All of them include basic health services: curative and prenatal care, deliveries, and vaccination. The health centre is managed by a nurse, usually assisted by a community health worker, a treatment seller and when possible a midwife or a matron, and trainees. At the time of the study, the diagnosis of malaria was based on clinical signs only as parasitological confirmation by thick smear or rapid test could not be performed.

#### Analysis methods

Records from the three health care facilities provided data about clinical diagnosis and anti-malarial prescriptions. They were filled in routinely by the nurse during consultations with patients and included only outpatient cases. Registers covering the period 1992–2004 were collected retrospectively from the health centres and entered on dBase IV software (dataBased Intelligence, Inc., Vestal, NY, USA) by the data entry staff in Niakhar and Dakar. The analysis was performed using Stata 8.0 software (StataCorp LP, Texas, USA).

## Results

Between 1992 and 2004, the total number of prescribed treatments was 292,965, corresponding to 110,093 patients. Among all treatments, 73,779 were anti-malarial drugs (25.2%).

### Anti-malarial prescriptions

**Figure 1:**
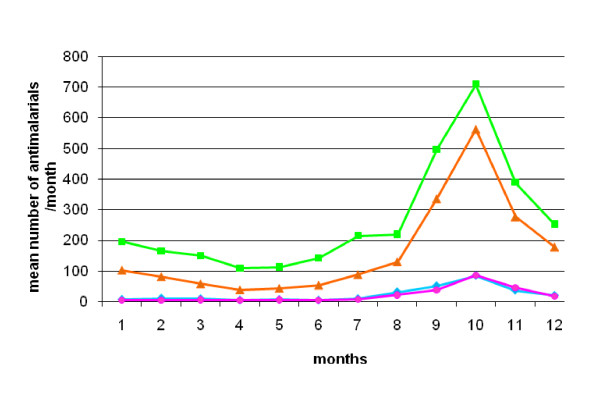
**Seasonality of anti-malarial prescriptions in health care facilities, Niakhar area, Senegal (1992–2004)**. Green, orange, blue and pink lines represent the mean number of prescriptions per month between 1992 and 2004, concerning chloroquine (CQ), quinine (Q), sulphadoxine-pyrimethamine (SP) and amodiaquine (AQ) respectively.

Anti-malarials were prescribed to 53,391 patients (48.5%), although malaria was clinically diagnosed in only 39,998 (74.9%) of them (Table 1). Anti-malarials were prescribed to 92.5% of patients with presumptive malaria and to 19.8% of patients with other diagnoses. Between 1992 and 2004, the monthly average number of prescribed anti-malarials was 461, versus 1,406 in the month of October alone. Sixty-two percent of all anti-malarials were prescribed during the four month-period of the rainy season (August to November) (Figure 1).

**Table 1:** Anti-malarial prescriptions to all patients, presumptive malarial and non-malarial patients.

	no of prescribed AM*	*%*	no of patients receiving AM	*%*	malarial patients	*%*	non-malarial patients^§^	*%*
total AM*	73 779	*100*	53 391	*100*	39 998	*74.9*	12 656	*23.7*
*CQ*^1^*(total)*	41 103	*55.7*	41 103	*77.0*	29 452	*71.7*	11 058	*26.9*
*Q*^2^*(total)*	25 546	*34.6*	25 546	*47.8*	23 783	*93.1*	1 570	*6.1*
*CQ+Q*^3^	16 391	*22.2*	16 391	*30.7*	15 608	*95.2*	717	*4.4*
*SP*^4^*(total)*	3 785	*5.1*	3 785	*7.1*	3 275	*86.5*	487	*12.9*
*AQ*^5^*(total)*	3 335	*4.5*	3 335	*6.2*	2 845	*85.3*	487	*14.6*
*AQ+SP*^6^	1 828	*2.5*	1 828	*3.4*	1 658	*90.7*	168	*9.2*

*AM: Anti-malarials, ^§^patients with unknown clinical diagnosis are excluded.

^1^CQ (total): total number of chloroquine treatments (CQ prescribed alone and CQ+Q), ^2^Q (total): total number of quinine treatments (Q prescribed alone and CQ+Q), ^3^CQ+Q: CQ and Q prescribed together (Q as first treatment and CQ as relay treatment), ^4^SP (total): total number of sulphadoxine-pyrimethamine treatments, ^5^AQ (total): total number of amodiaquine treatments, ^6^AQ+SP: bi-therapy with AQ and SP.

#### Chloroquine

**Figure 2:**
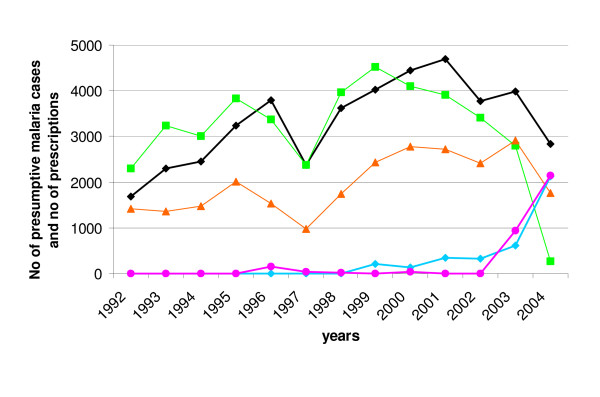
**Evolution of presumptive malaria cases and anti-malarial prescriptions in health care facilities, Niakhar area, Senegal (1992–2004)**. The black line represents the annual number of presumptive malaria cases. Green, orange, blue and pink lines represent the annual number of prescriptions of chloroquine (CQ), quinine (Q), sulphadoxine-pyrimethamine (SP) and amodiaquine (AQ) respectively.

CQ was prescribed to 41,103 patients (77.0% of patients who received anti-malarials), of which 29,452 (71.7%) had clinical malaria. It represented 55.7% of the prescribed anti-malarials. Figure 2 shows the evolution of CQ prescriptions from 1992 to 2004. CQ was given to an average of 3,162 patients per year, with differences according to the period of study.

From 1992 to 1996, CQ prescriptions rose progressively, in parallel with the diagnoses of clinical malaria. In 1997, a drop in both indicators occurred; then from 1998 to 2000, morbidity and prescriptions increased again. From 2000 to 2004, there was a dissociation between presumptive malaria morbidity and CQ prescriptions: the number of clinical malaria cases continued to increase, while CQ prescriptions started to decrease. Then, the number of malarial patients also declined and the drop in CQ prescriptions became steeper (in 2001–2004).

#### Other anti-malarial prescriptions

Quinine injections accounted for 34.6% of all prescribed anti-malarials; AQ and SP represented 4.5% and 5.1% respectively. Halofantrine was prescribed to six patients. Among the 53,391 patients treated by anti-malarials, 47.8% received quinine, 6.2% AQ and 7.1% SP; 3.4% received the AQ/SP bi-therapy (Table 1). Quinine prescriptions followed the same tendency as CQ until 1999. When CQ prescriptions started to decrease from 2000 to 2004, the use of quinine remained high and followed clinical malaria morbidity.

Concerning AQ and SP, a few prescriptions of either drug occurred sporadically before 2003 (e.g. AQ in 1996 and SP since 1999), but they were mainly prescribed together as a bi-therapy from 2003 onwards, following the new national policy. Overall, the rise of other anti-malarials prescriptions (quinine, SP) was concomitant with the decline in CQ use.

### Evolution of chloroquine prescriptions in health centres according to chemoresistance in the study area

**Figure 3:**
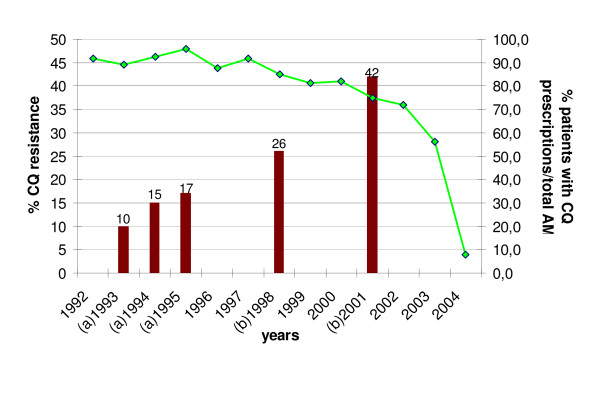
**Evolution of chloroquine resistance rate and proportion of patients with CQ prescriptions between 1992 and 2004 in Niakhar area**. Green line represents the proportion of patients receiving CQ prescriptions among all patients being prescribed anti-malarials (AM) (%). Red bars represent the rate of CQ resistance (%). (a) 1993, 1994, and 1995 estimates: *in vivo *chemosensitivity studies conducted in Diohine village, Niakhar area, Fatick region [16]. (b) 1998 and 2001 estimates: *in vivo *studies conducted in Kaolack sentinel site, 50 kilometres from Fatick [17].

Figure 3 presents the trends in the proportion of patients receiving CQ prescriptions among all anti-malarials between 1992 and 2004. From 1992 to 1997, the proportion of patients treated with CQ remained high (on average, 91% of patients receiving anti-malarials), then it decreased slightly from 1998 to 2000 (on average, 83%). The relative decrease of CQ prescriptions was more marked from 2001 and it confirms the tendencies displayed on Figure 2.

In parallel, there was an increase of chemoresistance in the region. Thanks to a careful surveillance of the area, the absence of resistance to CQ seemed proven until 1990 [22]. Although no suspicion of resistance was mentioned in the area of Niakhar, appearance of resistances or therapeutic failures of malaria to this drug in other areas of Senegal led to estimate chemosensitivity by relevant surveys. During the period of study, *in vivo *and *in vitro *tests showed a gradual increase of the resistance of *P. falciparum *to CQ in the area, going from 10% in 1993 to a maximum of 42% in 2001 [16,17,23,24].

### National policy change and anti-malarial treatment according to health care facilities

The new bi-therapy (AQ/SP) replaced CQ in health care facilities between 2003 and 2004. In 2004, overall CQ use accounted for 271 out of 3,370 (8%) patients being prescribed anti-malarial drugs, but figures varied considerably between dispensaries. In Toucar, no CQ was used that year, whereas 48 patients (6.4%) were still prescribed CQ in Ngayokhem, predominantly concentrated at the beginning of the year from January to March, and 223 (39.4%) in Diohine, distributed throughout the year.

The evolution of AQ/SP bi-therapy the same year also showed various results: overall prescription accounted for 1,653 patients in 2004 (49.1% of patients receiving anti-malarials), ranging from 3 (0.5%) in Diohine to 127 (17.0%) in Ngayokhem and 1,523 (74.1%) in Toucar.

Concerning quinine, 1,766 patients (52.4%) received injections in 2004: in Ngayokhem the majority of patients being prescribed anti-malarials were treated with quinine (525/749; 70.1%), 284 out of 566 (50.2%) in Diohine, and 957 out of 2 055 (46.6%) in Toucar.

In 2004, AQ and SP were still used as monotherapy in respectively 502 (15%) and 468 (14%) patients. SP was prescribed alone to 43% of patients receiving anti-malarials in Diohine and 25% in Ngayokhem. In Toucar, AQ monotherapy was delivered to 20% of patients who were prescribed anti-malarials.

## Discussion

As this observational study was held on a highly populated and closely observed demographic study site, it allowed the collection of data on more than 100,000 patients over 13 years, when important changes in malaria control policy were undertaken.

Anti-malarials represented 25% of all prescribed treatments and concerned nearly half of the patients consulting in the health care facilities. Among these treated patients, one fourth was not clinically diagnosed as malaria.

### Limits of the method: reliability of data

Data rely on information reported on health records. These registers are usually filled in by the nurse during outpatient's consultation. They are useful for clinical follow-up of the patients and for public health statistics but they are not adapted to research purposes. This lack of data precision is inherent to retrospective studies, which are based on passive data collection conducing to inevitable loss of information. During analysis, inconsistent and missing data had to be dealt with. Missing data may either come from the record itself, when no treatment has been mentioned by the nurse, or may be due to errors in data entry.

Data about malaria morbidity refer only to clinically diagnosed patients attending health centres, as confirmation of suspected cases was not routinely realized in peripheral health care facilities. Even though these presumptive malaria cases certainly overestimate real malaria morbidity, it is probably not related with the changes in trends during the time of observation and one may assume that overestimation was constant over the period.

### Interpretation of data

#### Overall trends in anti-malarial prescriptions

A large proportion of patients were treated with anti-malarials, although malaria was not confirmed parasitologically in most cases. This was due to the policy regarding management of patients in malaria endemic regions, before the introduction, in 2007, of rapid diagnostic tools to confirm malaria diagnosis. Until 2003, management of the patients consisted in early diagnosis and treatment at the health care facility or home-based treatment of fevers with CQ. In health centres, suspected cases of uncomplicated malaria were treated with CQ whereas quinine had to be saved for severe cases. Actually, health agents did not always comply with official guidelines and anti-malarials were extensively used. Frequently, patients suffering from another disease received anti-malarial drugs as well (19.8% in this study), usually CQ, as a "precautionary principle". Besides, quinine was not used for severe cases only.

Even if anti-malarials were prescribed throughout the year for a great number of patients, their use was mainly concentrated during the rainy season. Seasonality of prescriptions followed malaria morbidity patterns in this mesoendemic area, where malaria transmission occurs principally in a four-month period, leading to a rise in malaria cases between August and December, with a peak in the month of October. Presumptive malaria cases represented approximately 40% of total attendance through the observed period.

After a period of increasing clinical malaria cases and CQ prescriptions from 1992 to 1996, a sudden drop of prescriptions was observed in 1997, following the fall in consultations the same year in the health care facilities (Figure 2). Two explanations are proposed. First, rainfall was lower in 1997 than in the previous years (419 mm precipitations *vs *a yearly average of 522 mm during 1992–1996, as recorded in the Niakhar station by our own rain gauges), which may have reduced malaria transmission and, therefore, the morbidity attributable to malaria. Second, the immunization trials conducted until 1997 may have played a motivating role on the sanitary activities during the preceding years [21,25] inducing a high level of prescriptions. Other drug prescriptions also decreased by 10–20% between 1996 and 1997, associated to a decline in medical consultations.

Until the late 1990s, health workers did not change their practices because either they have not realized there was a rise in CQ resistance, as CQ decreasing effectiveness was not obvious, or they have lacked other therapeutic options. Besides, an additional study (Munier *et al*, *in press*) showed an absence of significant increase in patients' returns to the health centre after anti-malarial treatment over the period of study, which is not in favour of a perception of CQ decreasing effectiveness at the dispensary level.

In the early 2000s, the slight decrease in CQ prescription was concomitant with the persistence of the use of quinine at a high level and the rise of SP prescription. It could be attributed to a better awareness of the growing resistance of *P. falciparum*, which may have become clinically apparent, prompting health agents to opt for alternative drugs (SP or quinine). Another hypothesis is that health care providers anticipated the implementation of the new policy. Being aware of discussions held at national and local levels about the change of anti-malarial strategy, they may have started to change their practices before the new policy was adopted. Finally, patients' complaints, such as itching and eye allergies, intensified after the National Pharmacy liberalized the CQ sources of supply, possibly due to the presence of different excipients in these products that would be less tolerated by patients. This may have incited prescribers to use alternative drugs. At that time, they could also order cheaper generic SP at the health district (Maloxine^®^), compared to the Fansidar^® ^proprietary drug.

Furthermore, contrary to CQ, interest in quinine remained stable in this area. As it is known to be very effective, patients often request injections and they even seek them from other providers, when they cannot get them at the dispensary [20]. Thus health agents made an excess use of quinine.

As the delivery of drugs to the facility was dependant on the drug policy, the new directive was followed by the abrupt drop of CQ prescription in 2003–2004 (Figures 2 and 3) and the use of AQ and SP prescribed in association.

#### Therapeutic practices according to the health care facility

Beyond the general tendency, use of anti-malarials was highly variable according to the health centre, indicating distinct therapeutic practices, especially in 2004 after the change in national anti-malarial policy.

After the policy change, the National malaria control programme recalled the remaining CQ stocks from public health centres. However, some facilities finished their own CQ stock before ordering the new anti-malarial drugs. In any case, CQ was not supposed to be sold anymore at the district pharmacy's level. While in Toucar the national recommendations were followed thoroughly and CQ delivery was stopped in 2004, the delay was higher in the two other centres. In Ngayokhem, it seems that there were remaining stocks of CQ, which were further used during the dry season. The new efficacious drugs started to be prescribed in the rainy season, when a high increase of malaria morbidity was expected. Finally, in privately-run Diohine, CQ was still prescribed in 2004 all year long, although quinine prescriptions rose above CQ during the rainy season. It may be explained by the continuing supply of CQ by a catholic organization, which had not terminated its stocks. Only three patients received AQ/SP bi-therapy. In Toucar, the attendance of the same experienced nurse since 1996 is most probably a key factor in the respect of treatment guidelines.

Other practices outside official health care facilities, such as self-medication and purchase of medicines via the parallel drug market may have played a role. Previous socio-anthropological studies conducted in this rural zone [26-28] have shown that these practices are associated with complex behaviour resulting in a high proportion of patients not attending modern health facilities. Even though self-medication is generally the first response in case of a child's fever, populations preferably use symptomatic than etiological treatment [26]. They also considered that CQ was more efficient in preventing than treating malaria. A population-based survey conducted in the same area in 2001 [28] showed that anti-malarials represented only 18.2% of total self-medication (versus 64.7% for antipyretics). Furthermore, CQ was seldom available on markets in the years preceding the policy change, even though it was a cheap and affordable drug. Actually, customers rarely asked about it, and for the retailer, CQ was not profitable enough, being useful in a three-month period only. To cure malaria, they rather sold paracetamol or aspirin, which would treat the fever symptom (Le Hesran and Baxerres, personal communication).

This health-seeking behaviour was present during the entire period of study and probably does not interfere with overall trends in anti-malarial prescriptions in health care facilities, which represent the main anti-malarials providers in this area.

Neither AQ nor SP was found on the parallel market until 2003. Shortly after the policy change, it was noticed that some health workers in a neighbouring region [20] had not been properly trained for the new policy and did not always follow guidelines, prescribing AQ and SP in monotherapy, or quinine injections.

## Conclusion

Results reflected the overall level of CQ and other anti-malarial prescription in this area, and showed that health centres were the main providers of anti-malarials during the period 1992–2004.

Paradoxically, in spite of the emergence of CQ resistance in 1992, subjected to intense advertising in this highly-medicalized area where clinical trials were then conducted, no significant decrease in CQ prescription was observed between 1992 and 2000 and overall practices regarding malaria management changed slowly during this time. This fact could be explained by the absence of an obvious perceptible decrease in CQ effectiveness. Moreover, in the absence of guidelines from the Ministry of Health, therapeutic policy, stock clearance, and financial accessibility of CQ could be other plausible factors explaining this result.

These data also highlight the low reactivity between the appearance of chemoresistance and the change in national anti-malarial treatment policy. Given the cost of new anti-malarials was not affordable for a long time, morbidity and mortality figures were not yet alarming, and not to mention the poor administrative reactivity, change in national strategy took time to take off.

## Competing interests

The authors declare that they have no competing interests.

## Authors' contributions

AM participated in the conception of the study, collection and interpretation of data, and drafted the manuscript. AD contributed to the conception and implementation of the study, interpretation of data, and preparation of the manuscript. MC participated in the interpretation of data and revised the manuscript critically. ON participated in the supervision of the study and collection of data, and designed the data entry screen. PA contributed to the conception of the study, provided facilities and revised the manuscript. JPC was the conceptor of the study; he contributed to the analysis of data and to the preparation and review of the manuscript. JPC is guarantor of the paper. All authors read and approved the final manuscript.
